# The role of antiangiogenic agents in the treatment of gastric cancer: A systematic review and meta-analysis: Erratum

**DOI:** 10.1097/MD.0000000000007230

**Published:** 2017-06-08

In the article, “The role of antiangiogenic agents in the treatment of gastric cancer: A systematic review and meta-analysis”,^[1]^ which appeared in Volume 96, Issue 10 of *Medicine*, the figures and their legends appeared in the article incorrectly. They appear correctly below.

**Figure 2:**
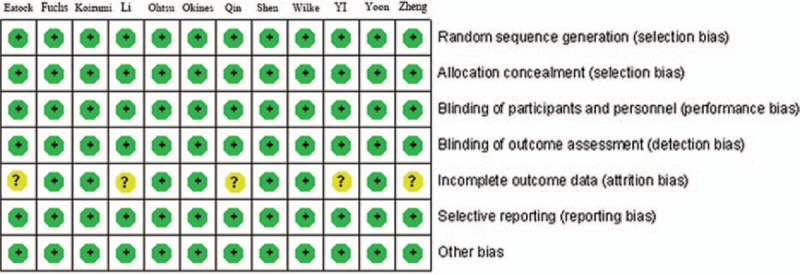
Evaluation of each study bias.

**Figure 3:**
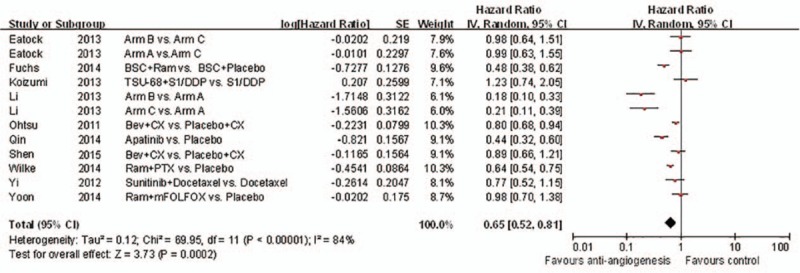
Forest plot and pooled HR & 95%CI for progression-free survival: Anti-angiogeneis agents of experimental versus control group.

**Figure 4:**
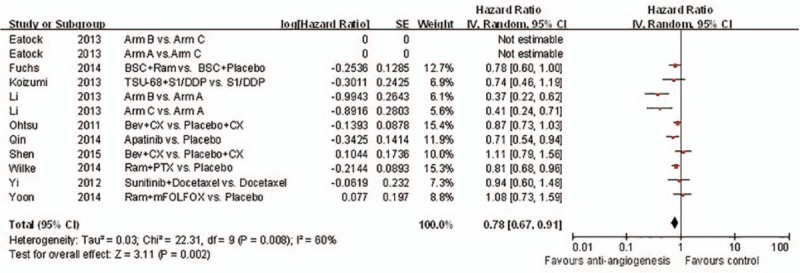
Forest plot and pooled HR & 95%CI for Overall survival: Anti-angiogeneis agents of experimental versus control group.

**Figure 5:**
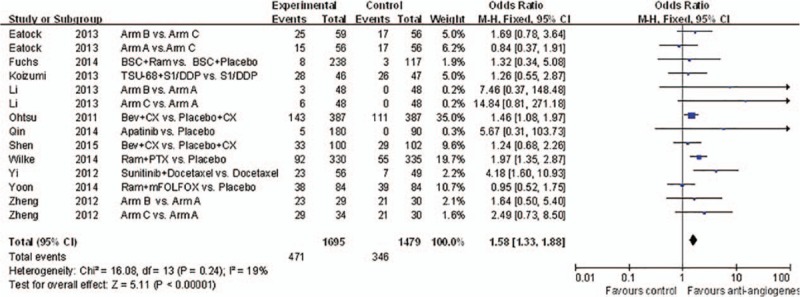
Forest plot and pooled HR & 95%CI for objective response rate: Anti-angiogeneis agents of experimental versus control group.

**Figure 6:**
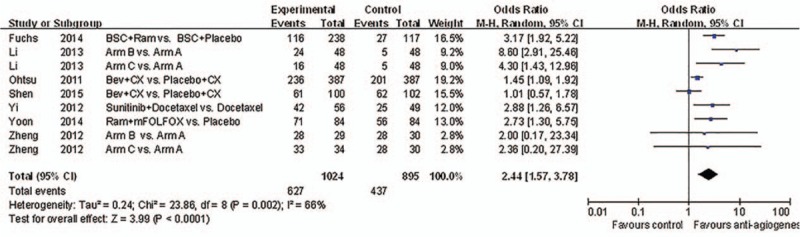
Forest plot and pooled HR & 95%CI for disease control rate: Anti-angiogeneis agents of experimental versus control group.

**Figure 7:**
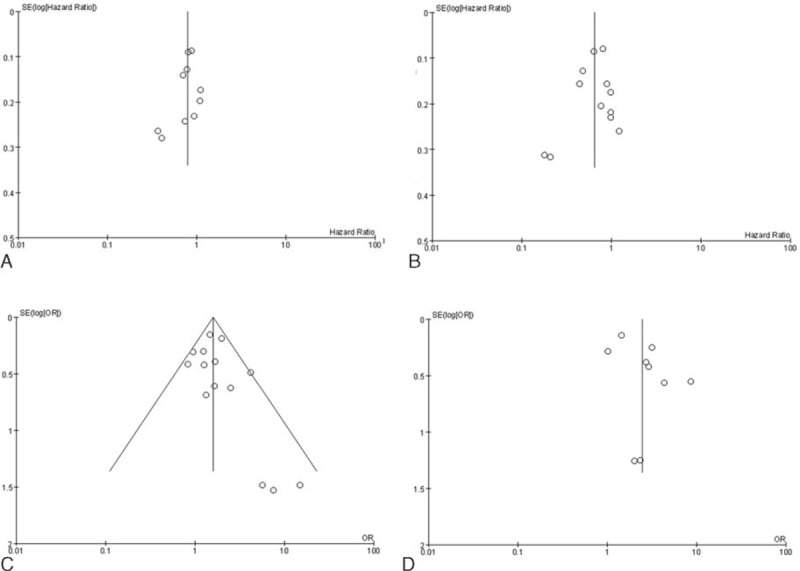
Publication bias qualitative analysis: funnel plot includes the meta-analysis of all studies outcome. (A) Overall survival, (B) progression-free survival; (C) objective response rate; (D) disease control rate.
